# The Role of Agency and Threat Immediacy in Interactive Digital Narrative Fear Appeals for the Prevention of Excessive Alcohol Use: Randomized Controlled Trial

**DOI:** 10.2196/32218

**Published:** 2022-06-13

**Authors:** Hendrik Engelbrecht, Laura Nynke van der Laan, Renske van Enschot, Emiel Krahmer

**Affiliations:** 1 Tilburg School of Humanities and Digital Sciences Tilburg University Tilburg Netherlands

**Keywords:** young adults, college students, alcohol abuse, drinking, EPPM, fear appeals, agency, serious games

## Abstract

**Background:**

Serious games for the training of prevention behaviors have been widely recognized as potentially valuable tools for adolescents and young adults across a variety of risk behaviors. However, the role of agency as a distinguishing factor from traditional health interventions has seldom been isolated and grounded in the persuasive health communication theory. Fear appeals have different effects on intentions to perform prevention behaviors depending on the immediacy of the consequences. Looking into how to increase self-efficacy beliefs for health behavior with distant consequences is the first step toward improving game-based interventions for adverse health outcomes.

**Objective:**

This study aimed to investigate the effect of agency on self-efficacy and the intention to drink less alcohol in an interactive digital narrative fear appeal. Furthermore, the communicated immediacy of threat outcomes was evaluated as a potential moderator of the effect of agency on self-efficacy.

**Methods:**

A web-based experimental study was conducted with university students (N=178). The participants were presented with a fear appeal outlining the consequences of excessive alcohol use in a fully automated web-based interactive narrative. Participants either had perceived control over the outcome of the narrative scenario (high agency) or no control over the outcome (low agency). The threat was either framed as a short-term (high immediacy) or long-term (low immediacy) negative health outcome resulting from the execution of the risk behavior (drinking too much alcohol).

**Results:**

A total of 123 valid cases were analyzed. Self-efficacy and intention to limit alcohol intake were not influenced by the agency manipulation. Self-efficacy was shown to be a significant predictor of behavioral intention. The immediacy of the threat did not moderate the relationship between agency and self-efficacy.

**Conclusions:**

Although agency manipulation was successful, we could not find evidence of an effect of agency or threat immediacy on self-efficacy. The implications for different operationalizations of different agency concepts, as well as the malleability of self-efficacy beliefs for long-term threats, are discussed. The use of repeated versus single interventions and different threat types (eg, health and social threats) should be tested empirically to establish a way forward for diversifying intervention approaches.

**Trial Registration:**

ClinicalTrials.gov NCT05321238; https://www.clinicaltrials.gov/ct2/show/NCT05321238

## Introduction

### Background

#### Overview

Excessive alcohol abuse is an alarmingly large cause of death in European Union member states, as well as in Norway, Switzerland, and the United Kingdom [1]. This is especially the case among young adults, with 1 in 4 deaths between the ages of 20 and 24 years occurring because of alcohol-related illness or injury. College students, who make up a large part of this age group, are often confronted with the social facilitation of alcohol use, leading to problematic drinking behaviors [2]. Given the prevalence and risk of excessive alcohol use, prevention-focused approaches should receive more attention to educate and train young adults to moderate their drinking. Fostering the execution of preventive behaviors demands the development of interventions that are effective in eliciting the desired change in behavior to avert short-term (eg, alcohol poisoning) and long-term adverse health outcomes (eg, liver disease) of excessive alcohol use.

We argue that the use of interactive digital narratives (IDNs) could be a promising tool to enable the training of preventive behavior through direct interaction with the narrative as a protagonist. In contrast to traditional passive narratives, an IDN involves computer-based interactive storytelling that allows the user to intentionally influence a nonlinear narrative. This means that the interactor is not only experiencing fictional reality but can also be part of it by being able to make meaningful decisions that affect narrative outcomes [3]. What sets serious games apart from more traditional passive forms of media is the opportunity to interact with and affect what happens in the game, making it important to establish the effect of interaction on outcomes that are relevant to serious game interventions. This study aimed to connect the theoretical construct of agency of interactors as the perceived meaningfulness of interaction with self-efficacy beliefs about health behaviors for immediate and long-term health threats.

#### Fear Appeals

Fear appeals have been widely assessed and used by many health communication researchers for interventions that focus on behavioral changes. Fear appeal communication aims to stimulate behavior change through the provision of a threat that causes a fear response. The message receiver is assumed to be motivated to resolve the arousal caused by the fear response and is presented with a solution to avert the threat. If the appeal is successful, the message perceiver adopts the prevention behavior. The term *prevention behavior* is used to describe the behavior needed to avert a health threat. For example, in the case of messages promoting vaccinations to prevent an infection with a disease, receiving the vaccination constitutes prevention behavior.

Fear appeals are commonly used in many public health campaigns, making use of mostly text-based or pictorial stimuli to elicit fear responses. One of the most widely applied, as well as well-researched, theories on the underlying process of fear appeal communication is the extended parallel process model (EPPM) [4]. The core assumption of the EPPM is the processing of threat information as either eliciting a fear control process (ie, the mitigation of threat-related processing through reactance) or a danger control process, which results in the adoption of threat prevention behavior. A meta-analysis on the effectiveness of EPPM-based fear appeals in the general health domain showed positive effects on behavior (as well as attitudes and intentions), with this effect being increased by the use of messages perceived as high in severity and susceptibility [5]. In other words, the success of the intervention is determined by first appraising the threat as serious and personally relevant (severity and susceptibility) and, second, seeing the prevention behavior as effective in averting the threat (response efficacy) and believing that one is capable of executing the prevention behavior (self-efficacy). The perception of self-efficacy has been shown to be a core determinant of fear appeal effectiveness in influencing the execution of prevention behaviors when severity and susceptibility are high [6].

Witte [4] posited that high *self-efficacy* means that a person needs to believe that he or she has the ability to execute a response that is recommended to him or her in the context of a fear appeal (ie, preventive health behavior). According to the EPPM, perceived *efficacy* (*self-* and *response efficacy*) communicated in a fear appeal determines whether a fear message is accepted. It is important to note that the evidence for the abovementioned effects does not rely solely on studies dealing with the health threat of alcohol exclusively but rather encompasses studies across a variety of risk behaviors such as smoking, drug use, and risky driving. Previous work supports the notion that fear appeals, specifically those addressing alcohol use among young adults, can raise efficacy and affect intention to drink alcohol (see the studies of Stainback and Rogers [7] and Moscato et al [8]); however, work in this domain remains scarce. Moreover, studies on the assessment of interactive narrative interventions in this area are nonexistent.

#### Narrative Appeals

Fear appeals used in public campaigns often make use of simple narratives through pictures and textual information that are aimed at persuading the message receiver by providing a way of averting the threat (ie, increasing efficacy beliefs). These simple narratives (eg, a picture of a man losing his leg because of smoking) heighten the relevant affective responses for persuasion (ie, fear and compassion), leading to more persuasive fear appeals [9]. Although these simple narratives are seen as more personally relevant than pure factual information, they are limited by the brevity of the narrative content. Although concise messages and pictorial stimuli engage narrative processing of the content [10], for more elaborate narrative appeals, the difference lies in the elaboration of the threat and solution. The additional context of narrative content strengthens transportation and identification through a heightened loss of self-awareness [11]. Evidence for this can be found in many studies showing narrative persuasion through heightened identification with the protagonist and transportation into the story world [12-14]. The term *story world* describes the structure surrounding the events of the story, which includes the locations and persons relevant to the events taking place [15]. Transportation into the story world plays an important part in narrative persuasion as higher transportation can result in affective and cognitive responses that are consistent with what the protagonist in the story world experiences [16].

In their review of narrative interventions, De Graaf et al [17] concluded that further research on how content is embedded into the narrative is needed. More specifically, they pointed to the integration of narratives into causal structures as an important way forward for health narratives. De Graaf et al [17] based this conclusion on the work by Dahlstrom [18], who showed that the integration of persuasive appeals as having an effect on character actions leads to lower resistance to persuasion. Although the aforementioned study was not conducted in the health domain, we can assume a possible transfer of this *causality effect* to interactive game-like formats. Although a passive narrative appeal can affect character intentions to enhance persuasion, an interactive appeal could potentially increase this effect by making the message receiver an active participant in the behavior addressed by the appeal. Earlier research clearly points to evidence supporting the effectiveness of narrative appeals, but there is little work on the potential influence of providing the message receiver with agency in interactive narrative appeals that strengthen the causal connection between prevention behavior and threat aversion.

#### The Role of Agency for Self-efficacy Beliefs

The term *agency* has been widely used for different forms of interaction with games and IDNs*.* In IDN research, agency is most commonly conceptualized as the perceived meaningfulness of action with regard to narrative outcomes. As argued by Murray [19] and other researchers in the field, control over narrative progression enables higher affective engagement, which is important for effective persuasion in narrative appeals [20]. According to Murray [19], agency is the “power to take meaningful actions and see the results of our decisions and actions.” This definition was extended by Tanenbaum and Tanenbaum [21]. They described the need for players to develop *competence* in how their actions affect the story world, and this need can be fulfilled through the provision of agency. This competence in learning that player actions have consequences (also called *procedural literacy*) on the story world is a necessity for meaningful interaction (ie, the operationalization of *agency*). For example, the ingestion of certain foods within the game world results in an increase in the (visible) weight of the main character.

Many investigations have provided interactive elements in their message design (see the studies by Winskell et al [22] and Carvalho et al [23]) but do not contrast interactive to passive appeals directly, making generalizations about the impact of perceived agency difficult. Only a few studies have compared interactive with noninteractive fear appeals, and these studies found contradictory results. The study by Panic et al [24] on children suggested that the interactive game used for the appeal distracted participants, thereby preventing the processing of the appeal content. In contrast, Kim et al [25] found that a game-based fear appeal resulted in stronger intentions to quit adverse behavior than a brochure. However, it has to be noted that these findings are hard to ground in assumptions of interaction alone, as the difference in the format of the appeal introduces more differences between conditions than interaction alone.

Although seldom investigated experimentally, operationalizing agency through meaningful interaction seems promising in how this could relate to self-efficacy for threat prevention behaviors. Through the experience of agency, prevention behaviors can potentially be trained, and self-efficacy beliefs can be strengthened. Agency itself is only concerned with the experience of meaningful interactions within the narrative, whereas self-efficacy is concerned with the transfer of beliefs about behaviors in the real world. Self-efficacy is defined as the belief of an individual that they can execute a behavior recommended by a fear appeal message [4]. In existing work, self-efficacy is sometimes used interchangeably with efficacy beliefs in the game itself, where the experience of meaningful interactions within the game is assumed to be related to heightened self-efficacy beliefs for this in-game behavior for the remainder of the game [26]. However, for this study, our focus is not on in-game efficacy but on the transfer of experienced agency to real-life self-efficacy beliefs.

Real-life self-efficacy beliefs are amenable through experimental manipulation in persuasive communication [6]; however, no previous study has investigated whether an interactive fear appeal that is high in agency increases real-life self-efficacy for the behavior executed in the game, as opposed to a passive appeal. The relationship between in-game agency and self-efficacy needs to be further grounded in the assumptions of social learning theory [27] to explain the potential transfer from in-game behavior to real-life efficacy beliefs. The social learning theory posits self-efficacy as an outcome of social learning processes. By using narrative experiences, behavior can be modeled through the observation of role models, which increases the salience of attitude formation in real-life contexts and increases self-efficacy on the part of the person interacting with the narrative [28]. Although observation alone contributes to *self-efficacy* beliefs, the effect is deemed greater for the execution of the target behavior by the person himself or herself. The interaction in an IDN can be hypothesized to increase *self-efficacy* through the development of competence in prevention behavior (as rooted in perceived *agency*).

In line with this, in this study, we expect that self-efficacy beliefs about real-life prevention behaviors can be affected by the provision of agency in an interactive narrative fear appeal. Although it is only a stepping stone in examining the process of persuasive IDNs for behavior change, the role of interaction as a key differentiator in the experience between passive and active media has rarely been investigated in a highly controlled narrative environment.

#### The Issue of Distant Threats

Although fear appeals for immediate threats have been shown to be effective, there is less evidence showing EPPM’s effectiveness for temporally distant threats [5]. For example, although smoking in adolescents has an impact on future health (*low immediacy*), interventions informing about immediate negative outcomes (*high immediacy),* such as bad breath, have been shown to be more effective in evoking behavior change [29]. This poses the question of how to design interventions that try to elicit prevention behaviors for threats that do not have immediate consequences or where immediate consequences are perceived as unlikely to occur.

As discussed by Klimmt and Hartmann [26], players are causal agents in IDNs, who derive their engagement with the game by experiencing *efficacy* from successfully conducting actions. The *immediacy* of a response causes temporal congruency between the cause and effect between player actions and game events. In the case of a cause-effect chain with high *immediacy*, there is little question on the part of the player with regard to their agency and, therefore, their self-efficacy to affect the story world. In terms of integrating a distant threat into an IDN, the narrative format allows the author to advance time in the story world, which opens up the opportunity to present players with an immediate causal connection between executed behavior and long-term consequences. Using this temporal flexibility of a narrative format, we hypothesized that the effect of agency on self-efficacy is moderated by the framing of the threat as either short-term (high immediacy) or long-term (low immediacy) adverse health outcomes.

### Goals of This Study

This study aimed to assess the effect of agency and threat immediacy on self-efficacy and, consequently, the intention to perform prevention behaviors. The following hypotheses were tested using an IDN in this study:

1. Hypothesis 1: Higher agency in the narrative progression of the IDN fear appeal results in higher perceived self-efficacy for prevention behavior.
2. Hypothesis 2: The effect of agency on self-efficacy is moderated by framing the threat as an adverse health outcome with either high or low immediacy.
3. Hypothesis 3: Higher perceived self-efficacy will lead to a higher intention to perform the target behavior.

## Methods

### Design

Data were collected from November 26 to December 18, 2020. The hypotheses, study design, and planned analyses were preregistered using the preregistration platform provided by the Wharton Credibility Lab. The independent variables were agency (low or high) and threat immediacy (low or high). Self-efficacy and behavioral intention served as the dependent variables in this study.

### Ethics Approval

This study was approved by the Research Ethics and Data Management Committee (REDC) of the Tilburg School of Humanities and Digital Sciences (TSHD) and was conducted as a 2×2 between-subjects experimental study (reference number: REDC 2020.141).

### Participants

A total of 178 participants were recruited from the Human Subject Pool of Tilburg University. All participants were enrolled in a master’s or bachelor’s degree program and received 0.5 course credits for participation in the study. Participants were allocated equally across all 4 conditions, and the study was conducted entirely on the web. No personally identifiable data (including IP addresses) were collected to ensure the anonymity of all participants.

Data were collected in 2 phases. The first half of the participants (95/178, 53.4%) who enrolled in the study were allocated to the high-agency condition. After the completion of data collection for high-agency participants, the second half (83/178, 46.6%) of the participants were assigned to the low-agency condition. The low-agency narratives were matched in terms of narrative content to the narratives of the high-agency group who took part in the study before them. All participants were randomly assigned to either the high- or low-immediacy conditions. Given the short time frame of data collection and the absence of events that could have an influence on drinking-related behavior (eg, public events), we assumed the time gap between the participation of the first and second halves of the participants to not affect our measures. Sufficient computer literacy and English language capabilities were assumed among the student population used in this study.

Participants were included in the analysis if they (1) were aged >18 years, (2) spent >480 seconds completing the survey, (3) passed both attention checks relating to narrative content, (4) correctly identified their experimental condition in the manipulation check, (5) did not choose to *drink an alcoholic drink* for all decisions in the interactive narrative, and (6) did not show signs of alcohol abuse. Participants were excluded if they were aged <18 years (2/178, 1.1%) or completed the questionnaire in an unreasonably short amount of time (14/178, 7.9%). In addition, participants were excluded if they failed 1 of the 2 attention checks, indicating that the narrative was not read attentively (21/178, 11.8%), or they failed the manipulation check asking them to recall whether they were presented with short- or long-term (high or low immediacy) consequences of excessive drinking (9/178, 5.1%). All participants who chose an alcoholic drink for all decision points in the narrative were excluded (3/178, 1.7%). Choosing to drink an alcoholic drink for all presented decisions would lead to the participants not executing the prevention behavior (choosing not to drink alcohol); therefore, they were also not presented with a self-efficacy statement. This means that participants would not be exposed to a full fear appeal, as they only received a severity, susceptibility, and response efficacy statement. As elaborated earlier, according to the EPPM, without self-efficacy, a fear appeal is unlikely to lead to the adoption of the proposed prevention behavior. Hence, 1.7% (3/178) of participants had to be excluded as they chose to drink alcoholic drinks for all the presented decisions. Finally, 3.9% (7/178) of participants were excluded because of signs of alcohol dependence as they scored >15 on the Brief Young Adult Alcohol Consequences Questionnaire (B-YAACQ) [36]. As the inclusion of participants with dependence would have skewed the results for the self-efficacy measure, it was decided to exclude them so that the results were more representative of a prevention-focused intervention applied to a healthy population.

The final sample used for analysis comprised 123 participants, with 59 (48%) in the high-immediacy condition and 64 (52%) in the low-immediacy condition. The split between high and low agency was almost equal to 49.6% (61/123) of participants in the high-agency group and 50.4% (62/123) of participants in the low-agency group.

The sample was representative of a student sample with a mean age of 21.4 (SD 3.086) years. Most held either a high school (53/123, 43.1%) or bachelor’s diploma (55/123, 55.3%), with few (2/123, 1.6%) participants having completed their master’s degree. There was a large gender imbalance in the sample, with approximately 72% (88/123) of the sample being female.

### Procedure

The entirety of this study took place on the web. Participants signed up through the Tilburg University Human Subject Pool. They were first presented with an information letter and then informed consent before agreeing to participate in the study. Next, demographic data (age, gender, and education) were collected, and participants were instructed to *imagine being in the place of the protagonist* for the entirety of the narrative. On the basis of their condition, they were presented with 1 of the 4 IDNs. After completion of the narrative, participants were asked to fill out several questionnaires. First, the 2 dependent measures—self-efficacy and behavioral intention—and the perceived fear measure were presented. Next, the participants had to fill in the attention check by answering 3 questions concerning the content of the narrative. This was followed by manipulation checks. Following this, severity, susceptibility, response efficacy, and perceived agency were measured. Finally, questions regarding disinhibition, perceived control over drinking, and frequency of drinking too much alcohol, as well as the alcohol dependence measure, were presented. After completing the questionnaires, participants were debriefed and offered to watch a video to potentially restore any adverse effects resulting from the study.

### Stimulus Material

#### Overview

The narratives showed a fictional scenario in which the participant was presented with a fear appeal message, together with a pictorial fear stimulus (including severity, susceptibility, and response efficacy statements). Pictures were added throughout the story to (1) improve transportation into the story world, which has been shown to improve narrative persuasion [12], and (2) strengthen the fear response when participants are presented with the threat during the fear appeal message presentation [31].

**Figure 1:**
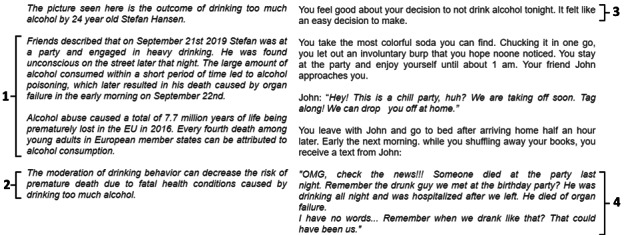
Excerpts from the narrative fear appeal comprising (1) severity and susceptibility, (2) response efficacy, (3) self-efficacy, and (4) threat avoidance messages in the high-immediacy condition.

The first part of the fear appeal message (Figure 1, left) was embedded into the narrative as a message that is displayed while the protagonist watches a video on the web during breakfast. The narrative subsequently presents participants with the risk situation—a house party—where the risk behavior—drinking alcohol—and, hence, the opportunity to perform the prevention behavior (declining a drink) is likely to be encountered. After completing the narrative scenario of the party, the protagonist of the story arrives at home. The last page of the narrative details the death of the protagonist or another person from organ failure, depending on whether the prevention behavior was executed by the participant (Figure 1, right). This event was described as having occurred either immediately after the party (high immediacy) or several months later (low immediacy). The entire narrative, as well as the decisions, were implemented in the survey using the survey platform Qualtrics.

The scenario of a house party was chosen as it is a familiar example to our target population and should, therefore, be easy to imagine and connect to real-life risk situations (to enable behavior transfer). Choosing organ failure as a threat made it possible to keep the threat congruent across different immediacy conditions while only changing the time frame. The threat needed to be severe to cause sufficiently high levels of perceived fear, whereas the pictorial stimuli needed to be congruent with the threat without invoking disgust. Disgust has been shown to potentially undermine appeal content when combined with fear stimuli [32]. The pictorial stimulus was taken from the Set of Fear Inducing Pictures developed by Michalowski et al [33] (picture index: *blood_60*) as part of the Nencki Affective Picture System [34]. This picture was chosen as it hides obvious features of the person and to avoid the influence of differences in identification with the patient and the moderate scores on valence and arousal around the midpoint in nonphobic individuals (as observed by Riegel et al [35]). According to Tannenbaum et al [5], the effectiveness of a fear appeal underlies a u-shaped curve of the relationship between the amount of induced fear and successful persuasion of the individual. To avoid potential reactance because of the evocation of an excessively high fear response, a moderately fear-arousing pictorial stimulus was selected.

#### Agency

A total of 4 different versions of the narrative fear appeal were created (1 per condition).

**Figure 2:**
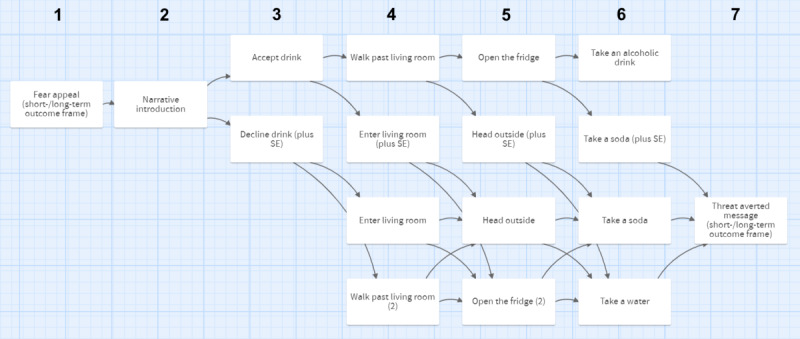
Overview of narrative nodes and progression for the high-agency condition. SE: self-efficacy.

As shown in Figure 2, agency was manipulated by giving participants choices at the end of every node (high agency) or by simply having them advance to the next page of the narrative by clicking the arrow button without making a decision (low agency). To be presented with the self-efficacy message in the high-agency condition, the participant had to execute the prevention behaviors (turning down an alcoholic drink) at the fictional party. To nudge participants to execute the prevention behaviors while retaining perceived agency, the participants were presented with 4 different decisions where they were able to decline or accept an alcoholic drink (Figure 2, nodes 2-5) using a foldback structure. This means that although the content presented to participants might differ slightly depending on the decision selected, the narrative would *fold back* to central events to enable the presentation of all participants with the same decision points. Once a participant declined the drink at any of the decision points, he or she was presented with a self-efficacy statement (Figure 2, row 2, denoted as *plus SE*) and was no longer able to choose options related to alcohol consumption. For example, a participant who accepted the alcoholic drink offer at the first decision point (Figure 2, node 2) will subsequently be presented with another 2 opportunities to turn down the alcoholic drink while the protagonist is making his way to the kitchen to obtain it. In decision 2, he can decide to walk past the living room and continue the pursuit of obtaining alcoholic drinks from the kitchen or decide not to drink that night (Figure 2, node 3) and enter the living room to chat with some friends who offer soft drinks. A participant who declines the initial drink offer for the first decision will still be asked to either enter the living room or continue toward the kitchen; however, these decisions (and all subsequent ones) will no longer be framed in terms of obtaining an alcoholic drink.

Every participant received only 1 self-efficacy message, as subsequent choices were no longer related to the prevention behavior. The ending of the story highlights the meaningfulness of turning down the drink by showing the adverse consequences that drinking could have had that night (Figure 1, section 4) while the protagonist arrived home safely because of the decision made by the participant. Participants who chose to drink an alcoholic drink at all 4 decision points (Figure 2, node 2-5) were presented with a different ending (Figure 2, top row, node 6) that detailed the death of the protagonist from alcohol poisoning (high-immediacy condition) or long-term alcohol use (low-immediacy condition). Participants in the low-agency condition were presented with a preset combination of nodes (without decision options) matched with the participants in the high-agency condition. Low-agency participants simply read the story and could not influence the decisions of the protagonist.

#### Immediacy

To manipulate outcome immediacy, the fear appeal at the beginning of the narrative was presented as organ failure because of excessive alcohol consumption over a short period (high immediacy) or continuous consumption of large amounts of alcohol over a period of months (low immediacy). Similarly, the threat aversion message (Figure 1, right side) was also adjusted to reflect either the aversion to a long-term or short-term health threat.

### Measures

#### Demographics

Demographic data were collected to account for appropriate randomization between the conditions. Participants were asked to indicate their age, gender, and educational background.

#### Dependent Measures

Self-efficacy beliefs were measured by adapting the item stems from Shi and Smith [36] to fit this study. Participants rated their perceived self-efficacy on 7-point Likert scales ranging from *strongly disagree* to *strongly agree* for 3 items. As the standards for what constitutes *too much alcohol* differ between individuals and are therefore difficult to specify in terms of the frequency of alcoholic drinks consumed [30], the items were formulated in terms of being able to *limit alcohol intake to low amounts.* For example, “I am able to limit my alcohol intake to low amounts.” Reliability analysis of the 3-item self-efficacy measure (mean 6.049, SD 0.988) indicated good reliability of the scale (Cronbach α=.882).

Behavioral intention was measured using items adapted from Fisher et al [37]. Although other studies have used a single-item probability question (eg, see the study by Carrera et al [38]), the approach by Fisher et al [37] uses contextual cues to assess behavioral intentions. As we believe the risk behavior to be especially likely to be exhibited in these situations, the contextual questions are more likely to reflect behavioral intentions rooted in real-life experiences. For example, “I intend to limit my alcohol intake to low amounts when being with friends.” The 3 items were scored on 7-point Likert scales from *strongly disagree* to *strongly agree*. The reliability analysis of the 3-item behavioral intention measure (mean 6.187, SD 1.193) indicated good reliability of the scale (Cronbach α=.882).

#### EPPM Measures

Items for the fear measure were taken from the Witte [4] recommendations for a different health threat. A total of 6 items were scored on 7-point Likert scales ranging from *not at all* to *very much*. The items asked participants about the intensity of affective response toward the message; for example, “How much did this message make you feel frightened?” Reliability analysis of the 6-item fear measure (mean 3.092, SD 1.357) indicated good reliability of the scale (Cronbach α=.898).

Items for perceived susceptibility and severity were adapted from Shi and Smith [36]. Although the items from Fisher et al [37] more closely resemble the items proposed by Witte [4], they do not explicitly connect the behavior and risk outcome for the response efficacy rating scales. Furthermore, Shi and Smith [36] used 3-item scales rather than the 2 items used by Fischer et al [37] and explicitly based their assumptions for their study on the Witte [4] EPPM rather than the focus on protection motivation theory used by Fisher et al [37].

Severity, susceptibility, and response efficacy items were measured with 3 items each on 7-point Likert scales from *strongly disagree* to *strongly agree*. As with the self-efficacy and behavioral intention measures, the measures are phrased in terms of either *drinking too much* or *drinking less* to account for individual differences in the perception of the appropriateness of alcohol intake.

The reliability analysis of severity (3 items; mean 6.333, SD 0.744; Cronbach α=.571), susceptibility (3 items; mean 2.832, SD 1.216; Cronbach α=.744), and response efficacy (3 items; mean 4.640, SD 1.308; Cronbach α=.792) showed moderate scale reliability.

#### Manipulation Check Perceived Agency

To validate agency manipulation, a scale was included to measure perceived agency after the participants experienced the narrative. The items for perceived agency were adapted from Fendt et al [39]. A question was dropped as it related to game enjoyment rather than perceived agency. The conceptualization of agency by Fendt et al [39] is congruent with the concept of agency applied in this study; for example, “I felt that the actions I took were meaningful within the context of the story.” Items were measured on 7-point Likert scales from *strongly disagree* to *strongly agree*. Reliability analysis of the 5-item perceived agency measure (mean 4.524, SD 1.522) indicated good reliability of the scale (Cronbach α=.881).

#### Manipulation Check Immediacy

To check whether participants perceived a difference in the immediacy of the presented threat, they were asked to recall the content of the threat message presented at the beginning of the narrative. Participants had to indicate whether the protagonist died because of *drinking too much frequently over a long period* (low immediacy) or *drinking too much at a party over a short period* (high immediacy).

#### Sample Characteristics

To account for potential individual differences between participants, disinhibition, drinking frequency, alcohol dependence, and perceived control over drinking behavior were measured. Behavioral inhibition, measured by a subscale of the Sensation Seeking Scale-V by Zuckerman [40], has been extensively linked to differences in arousal to media messages. Specifically, subscale 3 (*disinhibition*) has been shown to indicate that low sensation seekers exhibit stronger affective responses to fear appeals addressing alcohol use [41] and are highly correlated with the risk of heavy drinking behavior [42]. The items to measure disinhibition were taken unchanged from the scale by Lee and Shinn [41] but altered from 9-point to 7-point Likert scales ranging from *strongly disagree* to *strongly agree*.

The subscales for measuring frequency and perceived control regarding excessive alcohol consumption were taken from Carrera et al [38]. The items on perceived control were changed from a binary question format to Likert scale statements.

Frequency was measured by asking participants how often they *drink alcohol in excess* and was measured on a 7-point Likert scale ranging from *never* to *very frequently*. Perceived control was measured by 2 items asking participants whether they could *control* and *stop* drinking alcohol whenever they wanted. These 2 items were measured on 7-point Likert scales ranging from s*trongly disagree* to s*trongly agree*.

As alcohol dependence is difficult to measure accurately in student populations, the B-YAACQ was developed by Kahler et al [30] to measure the consequences of alcohol-related behaviors indicating dependence. An example item is “My drinking has gotten me into sexual situations I later regretted.” The B-YAACQ comprises 24 statements scored with binary yes or no answers. A score of >15 positive answers indicates alcohol dependence.

#### Analysis

**Figure 3:**
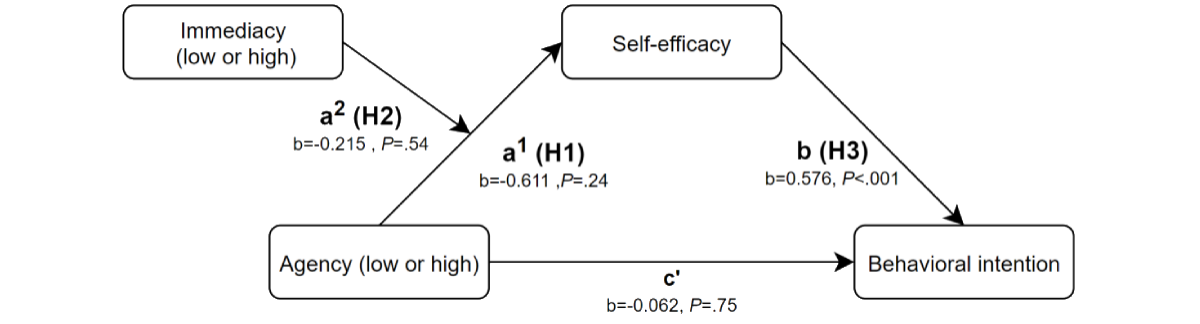
Moderated mediation model for the relationship between agency and behavioral intention. H:hypothesis.

The conceptual model shown in Figure 3 was analyzed using the PROCESS [43] plug-in in SPSS in conjunction with template mode 7 (moderated mediation model). This enabled the analysis of the direct effect of agency on self-reported perceived self-efficacy (hypothesis 1; path a^1^), moderation of the relationship between agency and self-efficacy by the outcome frame (hypothesis 2; path a^2^), effect of self-efficacy on behavioral intention (hypothesis 3; path b), and potential direct effect of agency on behavioral intention (path c′). A total of 5000 bootstrap samples were used for the analysis.

The a priori power calculation assumed a medium effect size (*f*=0.25) with the desired power level of 0.8 for our 2×2 research design. To reach adequate power, 32 valid samples were needed per condition.

## Results

### Deviation From Preregistration

For the main data analysis, a 2-way ANOVA was preregistered to test the effects of agency and immediacy on self-efficacy. However, we later realized that a more advanced model that also takes into account the possible moderating effect of immediacy provides a better fit for the data; hence, we report the analysis in the following sections. It should be noted that the preregistered ANOVA yielded essentially the same outcome.

### Randomization Check

A total of 123 cases were analyzed. We first examined our control variables to ensure that their scores were equally distributed across all 4 conditions.

Three 1-way ANOVAs were conducted to test whether group assignment had an effect on the perceived control over alcohol intake, inhibition, or age. We obtained no significant results for group membership on perceived control (*F*_3,119_=0.813; *P*=.49), inhibition (*F*_3,119_=0.531; *P*=.66), and age (*F*_3,119_=2.287; *P*=.08), indicating that the conditions did not have significantly different distributions of scores for alcohol intake, inhibition, and age. Chi-square tests were administered to ascertain the potential differences in the distribution of drinking frequency and gender across conditions. For drinking frequency, participants were grouped as nondrinkers, rare drinkers, or occasional and frequent drinkers. There were no significant differences in distributions across conditions for gender (N=123; *χ*^2^_3_=3.8; *P*=.28) or drinking frequency (N=123; *χ*^2^_6_=5.8; *P*=.44). In summary, no significant differences in perceived control over drinking behavior, inhibition, drinking frequency, age, and gender were found between the conditions. Thus, the randomization was successful.

### Manipulation Check

An independent-sample *t* test (2-tailed) was performed to check for the effectiveness of the agency manipulation by comparing the condition (low or high agency) with the perceived agency measures. There was a significant difference between the low-agency (mean 4.132, SD 1.596) and high-agency (mean 4.921, SD 1.342) conditions (*t*_121_=−2.965; *P*=.004), indicating an effective manipulation of agency.

### Hypothesis Tests

The analysis of path a_1_ indicates that the different levels of agency (low or high) are not significant predictors of the self-efficacy measure (path a_1_: *t*_119_=−1.182; *P*=.24; 95% bias-corrected and accelerated [BCa] CI −1.634 to 0.412), indicating that we had found no evidence to support hypothesis 1. There was also no interaction effect between agency and outcome frame as a predictor of self-efficacy (path a_2_: *t*_119_=.610; *P*=.54; 95% BCa CI −0.483 to 0.913). This indicates that the relationship between agency (low or high) and self-efficacy was not affected by the difference in outcome frames (high immediacy and low immediacy) presented to the participants (hypothesis 2). Looking at path b, we see that self-efficacy is a significant predictor of behavioral intention (path b: *t*_120_=5.135; *P*<.001; 95% BCa CI 0.354-0.798; hypothesis 3). Given that self-efficacy is the mediator in this model, agency did not have a direct effect on behavioral intention (path c′: *t*_120_=−0.325; *P*=.75; 95% BCa CI −0.437 to 0.314). Therefore, we observed no evidence to assume that the manipulation of agency and threat immediacy affected behavioral intention measures. An overview of these effects can be found in Table 1.

**Table 1:** Effects overview of the hypothesis tests for the moderated mediation model.

			Estimate (SE)		*t* test (*df*)		*P* value		95% CI
**Direct effects**									
	Agency→self-efficacy	−0.611 (0.517)		−1.182 (119)		.24		−1.634 to 0.412	
	Self-efficacy→behavioral intention	0.576 (0.112)		5.135 (120)		<.001		0.354 to 0.798	
**Interaction effect**									
	Agency×immediacy→self-efficacy	0.215 (0.352)		0.610 (119)		.54		−0.483 to 0.913	
**Total effects**									
	Agency→behavioral intention	−0.062 (0.189)		−0.325 (120)		.75		−0.437 to 0.314	

### Exploratory Findings

Additional exploratory analyses were conducted to contextualize the null effects. A median split was conducted by analyzing the effect of agency on self-efficacy for participants scoring high on susceptibility to ascertain whether the appeal or the pre-existing characteristics of the sample caused a potential floor effect for the susceptibility measure. The adjusted sample (75/123, 61%) showed no significant relationship between the agency and self-efficacy measures (*t*_73_=1.928; *P*=.06). Similarly, we evaluated whether a possible ceiling effect of self-efficacy measures might have influenced the overall effect of agency on behavioral intention. After conducting the median split, it was found that only a small number of participants (40/75, 53%) scored below the median, with a relatively large proportion scoring exactly on the median (24/75, 32%). Given the low variability in the sample and, thus, only a limited sample, it was decided not to conduct any further analysis on the remaining 32.5% (40/123) of participants.

## Discussion

### Principal Findings

Although the manipulation of agency was found to be successful, this study found no effect of agency on perceived self-efficacy, and this relationship was also not influenced by the framing of the threat as a short-term (high immediacy) or long-term (low immediacy) adverse health outcome. A significant effect was found for the relationship between self-efficacy and behavioral intention. To point forward for future studies, the null findings of this study need to be discussed with regard to their contribution by contrasting the different approaches taken for this study.

### Operationalization of Agency

In this study, agency had no significant effect on the self-efficacy perceptions of the participants. Agency was operationalized as the one-time execution of an action with a meaningful impact on the outcome of the narrative. To achieve this, a foldback structure was used to ensure that participants would encounter the same narrative content and be presented with a complete fear appeal where the prevention behavior was executed, and a self-efficacy message could be displayed. Although the manipulation check showed a difference in perceived agency between high- and low-agency conditions, the perceived agency might still not have been impactful enough to cause a change in the self-efficacy measure. Owing to the nonexistence of related work on the effects of agency on self-efficacy, these results are difficult to compare with other operationalizations of agency in the field. However, looking at the different theoretical conceptualizations of agency, we can derive possible explanations.

The manipulation in this study could conceivably be seen as a low-agency condition if agency is defined as the balance of affordances and constraints provided by the system [44]. Using the foldback structure increases constraints for affordances (ie, the number of choices) in our narrative, leading to less agency than using a system that provides more choices (ie, branches) to the user. It can be argued that agency is restricted to only one possible outcome in our narrative. According to Crawford [45], the degree of interactivity is determined by the functional significance of a choice and its perceived completeness. Perceived completeness means that the number of choices corresponds to the readers’ imagined number of possible choices. Although functional significance was high in this study, relating decisions back to story outcomes, perceived completeness was very limited. This highlights the antagonistic relationship of targeted interventions using a game-like format. With less authorial control over the story, the intervention can exert little control over the presentation of the message in terms of the content and chronology of persuasive aspects. This is especially the case when applying a health intervention framework (such as the EPPM), which segments the intervention into distinct parts.

Heightening perceived completeness could be achieved, given the successful use of illusory agency, presenting options that are perceived to be more complete even if they fold back to the same outcomes, or by loosening authorial control in more open experiences such as a sandbox game that enables emergent storytelling. The question is whether the elicitation of self-efficacy through agency is reliant on control over the chronology of message components or whether heightened agency through emergent storytelling can make up for the loss in control by providing a closer link between the protagonist and the message receiver in terms of perceived completeness of the available actions. Taking this a step further, there is evidence to form the assumption that agency does have an effect on persuasion, even if the agency manipulation is completely decoupled from the persuasion attempt and simply induced as a state before the intervention (eg, see the study by Damen et al [46]).

The wide range of used agency definitions and their heterogeneous operationalization make generalizations about agency effects in health interventions difficult. For future studies, the plural modality of agency should be taken into account [47] and tested experimentally to establish discriminant validity for different agency constructs. This means that not only the kind of agency needs to be taken into account but also how this can be operationalized most effectively, within or outside the intervention, to heighten the extent of perceived agency in participants.

### Immediacy and the Malleability of Self-efficacy Beliefs

In this study, we found no significant effect of threat immediacy on the relationship between agency and self-efficacy. Organ damage was chosen as the threat as it can be a result of either short-term or long-term risk behavior and was henceforth communicated as a consequence of a single night or multiple months of drinking. There is ample work evaluating the effect of fear appeals on one-time versus repeated risk prevention behaviors, showing that appeals advising the one-time execution of behaviors are more likely to succeed [5]. However, comparisons of short- and long-term outcomes in fear appeal threats are scarce, making it difficult to base the manipulation on existing work in the field. Although the manipulation checks ensured that participants noticed the manipulation, this might not have made the framing of threat immediacy salient enough to result in a significant effect on the relationship between agency and self-efficacy.

In most IDN research, as well as game studies, behavior transfer between digital and real-life behavior is often assumed to be high when the digital execution matches that of real-life analog behavior. The prevention behavior for the threat in this study does not constitute a one-time behavior, as it needs to be executed multiple times for the threat to be averted. With regard to immediacy in this study, there is, therefore, a mismatch between both our short- and long-term threats and our digital narrative in which the prevention behavior (rejecting a drink) is only executed once. Although a single appeal message can influence self-efficacy for repeated prevention behaviors (eg, see the study by Smith and Stutts [29]), in this study, a single execution of the behavior might not have been enough to affect self-efficacy beliefs. A possible explanation could be the depth of processing of the message itself, which hinders message acceptance and, therefore, undermines possible effects on short- and long-term threat prevention. Presentation with a single persuasive message activates the systematic processing of this message. Heuristic processing occurs only after repeated exposure, which breaks down resistance to persuasion and heightens fear appeal effectiveness [36].

A possible direction for future studies would be to make the long-term, as well as the short-term, impact of risk behavior more salient through the provision of repeated interventions. As discussed by Shi and Smith [36], EPPM-based fear appeals with long-term threats are more effective after 3 exposures than after a single exposure. It is unclear whether this also holds for short-term threats. Future research should investigate whether intervention repetition affects self-efficacy differently for threats with high and low immediacy.

### Lack of Susceptibility

The null effects for agency and threat immediacy could also result from the low perceived susceptibility of the target population (young adults) to the threat used in this study (organ failure because of excessive drinking). The so-called *optimism* concerning the likelihood of adverse health effects has been well established in other studies and potentially creates reactance during persuasive attempts for risk behaviors involving drinking, drunk driving, and smoking in young adults [48-50]. Organ failure is an extreme health threat that might be too abstract (ie, perceived as unrealistic) for young adults, leading to low perceived susceptibility. It can be argued that although organ failure from one night of binge drinking is immediate in a temporal sense, it is quite distant when it comes to the perceived likelihood of affecting young adults. This means that although general severity and response efficacy evaluations can be perceived as high, the link to personal relevance does not come into play because of low perceived susceptibility and the high baseline of perceived self-efficacy for this population (ie, optimism).

The issue of perceived threat susceptibility can be further explained by our exclusion criteria. The criteria specifically excluded participants who showed signs of alcohol dependence when they might actually have been participants for whom the threat would be most relevant and the preappeal efficacy would be low. However, as the aim is to increase the effectiveness of early intervention approaches, more effective ways of eliciting prevention behaviors for normal populations should still be investigated more thoroughly.

An alternative approach could be the use of appeals that do not focus on health threats to increase susceptibility as a prerequisite for successful narrative persuasion. Health threats themselves have been the main focus of health-related interventions and are effective for older cohorts; however, this might not always be the case for younger populations. As suggested by Pechmann et al [51], the use of social threats seems to be more effective for younger populations as they are more immediate than health threats are perceived, leading to higher perceived susceptibility. Therefore, it would be valuable to extend this line of research by trying to evaluate the effectiveness of appeals addressing short-term versus long-term social threats. As argued previously, the potentially weak cause-effect chain perceived by young participants could also potentially be strengthened through the use of social threats, which in turn might strengthen the effect of agency. If the threat is seen as relevant and the prevention behavior as effective, having participants execute the prevention behavior to avert a social threat might heighten self-efficacy, given high perceived agency over the narrative outcome.

### Methodological Limitations

This study did not use a pre- or postmeasures design to account for the potential influence of baseline beliefs already present in participants before the appeal message was presented. However, it has to be noted that disinhibition, drinking frequency, alcohol dependence, and perceived control over drinking behavior were measured to account for individual differences between participants. These were measured after the appeal message so as to minimize the influence on the responses to the IDN appeal. Although we believe that these constructs relate to either stable traits or general behavior and therefore should not have been influenced by the manipulation, we cannot fully discount the potential influence of the IDNs on these constructs.

Furthermore, it should be noted that the current sample was predominantly women (88/123, 72%). Previous studies have shown gender differences in the effectiveness of fear appeals, which might have influenced the results. In general, this sample might not be representative of the general population.

Finally, this study fell short of its goal of collecting a total of 140 valid samples, leading to a potential shortcoming in statistical power, given the complex study design. Furthermore, it could even be that the authors assumed a medium effect size for a single intervention transferring virtual behaviors to real-world beliefs and behavioral intentions.

### Contextual Factors

Finally, the context of this study must be mentioned. The study was conducted during the COVID-19 pandemic, which limited the participants’ exposure to risk situations and could have skewed the general perception of the appeal as personally relevant. Although there was no active lockdown in place at the time of data collection, there was a limit to the number of people allowed to gather more generally. This made the otherwise common risk environments that facilitate drinking, such as parties, almost nonexistent. As social facilitation of drinking is one of the core determinants of risk behavior related to excessive alcohol use for college students [2], the context during which the data collection took place could have affected self-efficacy beliefs because of nonexposure to the threat in real life. Considering the abovementioned limitations of this study, going forward, the findings should be replicated in a nonpandemic context to be generalizable for the target population.

### Conclusions

In this preregistered study, no significant effects were found for agency on self-efficacy and behavioral intention. In addition, we observed no effect for the influence of threat immediacy on the relationship between agency and self-efficacy.

The multitude of conceptual distinctions of different forms of agency and the different ways of integrating them into IDNs provides a challenge that needs further evaluation to affect self-efficacy and, more generally, behavioral change. There is a need to empirically test the difference between conceptually different agency concepts to establish their impact on persuasiveness in narrative health interventions. Although there is evidence to assume the effects of interaction on antecedents of narrative persuasion (eg, identification and transportation), more work is needed to understand how different agency conceptualizations directly affect the processing of health messages. In addition, more work is needed that empirically contrasts passive and interactive interventions to isolate the effects. Much of the previous work done in this field use fully-fledged serious game experiences without passive controls, making it difficult to ascribe effects to single factors in such complex systems. With regard to the theoretical assumptions underlying narrative fear appeals, the personal relevance of the content should be ensured through pilot testing before the deployment of the intervention. Without creating the personal relevance of the narrative fear appeal, the intended effects could be undermined because of a lack of perceived susceptibility to the threat. Understanding how different kinds of threats are perceived by different target populations should be investigated more thoroughly to adapt interventions more effectively. Furthermore, the effect of intervention repetition for different kinds of prevention behaviors is poorly understood, making generalizations across different fear appeal threats difficult. A systematic approach contrasting one-time and repeated prevention behaviors for different kinds of threats could establish a clearer picture of the potential necessity of adaptations for threats that are the result of repeated risk behaviors.

In conclusion, despite the null findings for the effects of agency on self-efficacy, this study highlights the potential for further exploration of agency concepts and threat types in terms of their embedding into a narrative intervention.

## Appendix

Multimedia Appendix 1 CONSORT-EHEALTH checklist (V 1.6.1).

## Abbreviations

BCa: bias-corrected and accelerated
B-YAACQ: Brief Young Adult Alcohol Consequences Questionnaire
EPPM: extended parallel process model
IDN: interactive digital narrative
